# American Journal of Pharmacy

**Published:** 1854-11

**Authors:** 


**AMERICAN JOURNAL OF PHARMACY. Edited by Prof. W. Proctor, of the Philadelphia College of Pharmacy.**

In calling the attention of our brethren to this work so valuable to the profession, and so honorable to this department of science in the country, we feel that we are discharging an obligation imposed upon us by our position. We feel too that it would be unnecessary to say more than that such a work is demanded by the wants of the profession, and that this periodical *meets* these wants. We have gleaned from its pages some valuable practical thoughts which we have profitably reduced to practice. We shall add to the interest of our Journal by freely extracting from it in future. We can cheerfully recommend it to the favorable consideration of the profession.

